# Invasion and trafficking of hypervirulent group B streptococci in polarized enterocytes

**DOI:** 10.1371/journal.pone.0253242

**Published:** 2021-06-15

**Authors:** Giuseppe Valerio De Gaetano, Germana Lentini, Roberta Galbo, Francesco Coppolino, Agata Famà, Giuseppe Teti, Concetta Beninati

**Affiliations:** 1 Department of Human Pathology, University of Messina, Messina, Italy; 2 Department of Chemical, Biological and Pharmaceutical Sciences, University of Messina, Messina, Italy; 3 Charybdis Vaccines Srl, Messina, Italy; 4 Scylla Biotech Srl, Messina, Italy; Universitatsklinikum Erlangen, GERMANY

## Abstract

*Streptococcus agalactiae* (group B streptococcus or GBS) is a commensal bacterium that can frequently behave as a pathogen, particularly in the neonatal period and in the elderly. The gut is a primary site of GBS colonization and a potential port of entry during neonatal infections caused by hypervirulent clonal complex 17 (CC17) strains. Here we studied the interactions between the prototypical CC17 BM110 strain and polarized enterocytes using the Caco-2 cell line. GBS could adhere to and invade these cells through their apical or basolateral surfaces. Basolateral invasion was considerably more efficient than apical invasion and predominated under conditions resulting in weakening of cell-to-cell junctions. Bacterial internalization occurred by a mechanism involving caveolae- and lipid raft-dependent endocytosis and actin re-organization, but not clathrin-dependent endocytosis. In the first steps of Caco-2 invasion, GBS colocalized with the early endocytic marker EEA-1, to later reside in acidic vacuoles. Taken together, these data suggest that CC17 GBS selectively adheres to the lateral surface of enterocytes from which it enters through caveolar lipid rafts using a classical, actin-dependent endocytic pathway. These data may be useful to develop alternative preventive strategies aimed at blocking GBS invasion of the intestinal barrier.

## Introduction

*Streptococcus agalactiae* or group B streptococcus (GBS) is a Gram-positive bacterium that can cause invasive disease, including sepsis and meningitis [1]. Neonatal GBS disease occurring during the first week of life is defined as early-onset disease (EOD) and is often initiated by aspiration of secretions from the genital tract of colonized mothers during parturition [2]. Disease occurring from day 8 to 89 after birth is defined as late onset disease (LOD) [2, 3]. EOD can manifest itself as sepsis, pneumonia and, less commonly, meningitis. In contrast, meningitis is frequent in LOD and can be followed by permanent neurological disorders [4]. GBS infection is increasingly detected in nonpregnant adults and in the elderly and is often associated with serious pathological conditions such as diabetes mellitus, cirrhosis, cancer and endocarditis [5, 6]. GBS frequently colonizes the gut and the genital tract and its ability to adhere to mucosal epithelial cells is an essential factor in these activities [2, 3]. Moreover, invasion of epithelial barriers is an obligatory event in pathogenesis [7, 8].

It has been proposed that the gut is a port of entry of GBS in LOD, which is frequently caused by strains belonging to the CC17 "hypervirulent" clone complex [9, 10]. Indeed, these strains often colonize the infant gut in the first three months of life and can be isolated also from newborns with GBS negative mothers [9]. CC17 strains express a unique set of adhesins and have been described as being particularly capable of invading the endothelial cells of the blood-brain barrier [10]. Their ability to interact with the gut epithelium has received comparatively less attention. In the intact gut mucosa, only the apical membranes of epithelial cells are exposed to the external environment, but, when damaged by microbial toxins or inflammation, lateral surfaces also became available for interactions with bacteria. Interestingly, basolateral cell membranes are more susceptible than apical surfaces to invasion by some bacteria, such as *Listeria monocytogenes* [11], *Shigella flexneri* [12] and *Campylobacter jejuni* but not by other enteropathogens, such as *Salmonella spp*. [13]. In the present study, we aimed to gain insights into the mechanism of invasion of enterocytes by hypervirulent GBS belonging to the LOD-associated CC17 clonal complex. To this end, we used the Caco-2 cell line that reproduces many features of the natural structure and physiology of the colonic epithelium [14, 15]. Our results indicate that GBS can efficiently invade polarized cells from their lateral surfaces by an actin- and caveolae-dependent mechanism.

## Materials and methods

### Bacterial strains and reagents

The GBS strain BM110 (serotype III, CC17) and *Salmonella enterica* serovar Typhimurium (strain M20) were used in the present study. They were grown at 37°C in Todd-Hewitt broth and Luria Bertani broth, respectively (both from Difco Laboratories). Rabbit polyclonal anti-GBS serum was obtained from animals immunized intravenously with formol-inactivated GBS. Rabbit polyclonal anti-EEA-1 antibodies (ab2900) and goat polyclonal anti-rabbit IgG Alexa Fluor 488-conjugated secondary antibodies (ab15007) were obtained from Abcam, Cambridge, UK. Lysotracker Red 500nM (L7526, Invitrogen) was used to label acidified endosomal compartments.

### Caco-2 culture

The human intestinal Caco-2 cell line (ATCC HTB-37; colorectal adenocarcinoma) was seeded in 24-well plates at a density of 1 x 10^4^ cells per well in RPMI-1640 medium (R8758, Sigma-Aldrich) supplemented with 10% (vol/vol) fetal bovine serum (1203C, Sigma-Aldrich) and penicillin-streptomycin (100 IU of penicillin and 0.1 mg/ml of streptomycin, P433, Sigma-Aldrich) and cultured at 37°C in a humidified 5% CO_2_ incubator. The culture medium was changed every three days and cells were used at 5 and 21 days after seeding. Confluence and cellular differentiation were monitored by optical microscopy and immunofluorescence. It was determined in preliminary experiments that Caco-2 cells show similar growth rates, general morphology and intracellular distribution of actin filaments when grown in RPMI-1640 medium supplemented with 10% fetal calf serum or in the medium recommended by ATCC, which contains 20% serum. Previous studies have shown the formation of fully differentiated monolayers with functional intercellular junctions in Caco-2 cells grown under the culture conditions used in the present study [16].

### Adhesion and invasion assays

Before each experiment, cultured Caco-2 cells were washed three times and infected with bacteria. Briefly, bacteria were grown to the mid-log phase, collected by centrifugation, washed and resuspended to the concentration of 1x10^7^ CFU/ml in pre-warmed serum-free RPMI. Then bacteria were added to 5-day-old sub-confluent islands or 21-day-old post-confluent monolayers at a MOI of 40. After 1h of incubation at 37°C in the presence of 5% CO_2_, extracellular bacteria were washed away by rinsing cultured cells three times with Dulbecco’s phosphate buffered saline without calcium and magnesium (DPBS; Euroclone S.p.A., Milan, Italy). The numbers of bacteria attached to the cells were measured by plating cell lysates on blood agar plates, as previously described [17]. To measure bacterial internalization, the infected cells were rinsed three times with DPBS and incubated for an additional 1 h in RPMI containing bactericidal amounts of penicillin and streptomycin (200 U/ml and 200 μg/ml, respectively) as previously described [18]. In some experiments we performed bacterial counts also at other times after infection, as indicated. Adherence and invasion values were expressed as percentage values using the formula: recovered CFU/initial inoculum CFU x 100.

### Disruption of intercellular junctions

Monolayers were washed and incubated with DPBS without calcium and magnesium for 1 h to disrupt intercellular junctions. In some experiments, cells were exposed to ethylene glicol-bis-(β-aminoethylether)-N, N, N’, N’-tetraacetic acid (EGTA) (E3889 Sigma-Aldrich) at a 10μM concentration in DPBS for 15 minutes prior to incubation with GBS to induce weakening of cell-to-cell junctions. Treatment with EGTA at this concentration (10μM) did not result in cellular detachment from the plate wells. Weakening of intercellular junctions after treatment with EGTA or Ca^++^-free medium was confirmed microscopically, as evidenced by gap formation in the monolayers.

### Fluorescence microscopy

Structured-illumination fluorescence microscopy analysis was performed using a Zeiss Observer.Z1 Apotome apparatus and images were acquired with the AxionVision software. Caco-2 cells were cultured on 18 mm^2^ coverslips at a density of 1 x 10^4^ cells per coverslips. Cell growth and confluence were monitored daily. To visualize bacterial adhesion, coverslips were washed at 1 h post-infection and fixed with 3.7% formaldehyde for 15 min at room temperature. After a 1 h blocking step with BSA (2% in DPBS) and several washes, coverslips were sequentially incubated for 1 h with rabbit polyclonal anti-GBS serum and with Alexa Fluor 488-conjugated secondary antibody both diluted 1: 2,000 in DPBS supplemented with BSA 1%. To visualize bacterial invasion, non-adhering bacteria were washed away and cells were further incubated with antibiotics to remove residual extracellular bacteria. Then coverslips were fixed and, after blocking with BSA, permeabilized with 0.1% Triton X-100 for 10 minutes. Phalloidin-iFluor 555 (ab176756, Abcam) and 4′-6-diamidino-2-phenylindole (DAPI) (D8417, Sigma-Aldrich) were used according to the manufacturer’s instructions to label, respectively, actin and DNA (both eukaryotic and bacterial DNA). At least 10 pictures were examined in each experiment.

### Endocytosis inhibitors

To study the mechanisms of GBS internalization, Caco-2 cells were treated with the following chemical inhibitors: chlorpromazine (CPZ; C8138-54, Sigma-Aldrich) and monodasylcadaverine (MDC; D4008, Sigma-Aldrich) for inhibition of clathrin-mediated endocytosis; methyl-β-cyclodextrin (MβDC; C4555, Sigma-Aldrich) and genistein (Gen; G6649, Sigma-Aldrich) for inhibition of cholesterol and tyrosine kinase, respectively, in lipid rafts; cytochalasin B (CytB; C6762, Sigma-Aldrich) and nocodazole (Ndz; M1404, Sigma-Aldrich), for inhibiting cellular actin and microtubules, respectively. Cells were pre-incubated with these compounds for 1 h prior to infection at the indicated concentrations. All infections were performed in presence of inhibitors, while control cells were incubated with vehicle only. Epithelial cell viability was evaluated by trypan blue staining after incubation with the inhibitors and was always > 90%.

### Statistical analysis

All experiments were repeated at least three times and the data were expressed as means ± standard deviations (SD). Data were analyzed for statistical significance by the Mann-Whitney or the one-way ANOVA tests. A p-value of 0.05 was used as the threshold for significance.

### Ethics statement

No *in vivo* studies were conducted. To obtain rabbit polyclonal anti-GBS serum, rabbits were immunized at the animal facilities of University of Messina, according to the European Union guidelines for the handling of laboratory animals. The procedure was approved by the local animal experimentation committee (Comitato Etico per la Sperimentazione Animale, permit 18052010).

## Results

### Adhesion of GBS to Caco-2 cells

**Fig 1:**
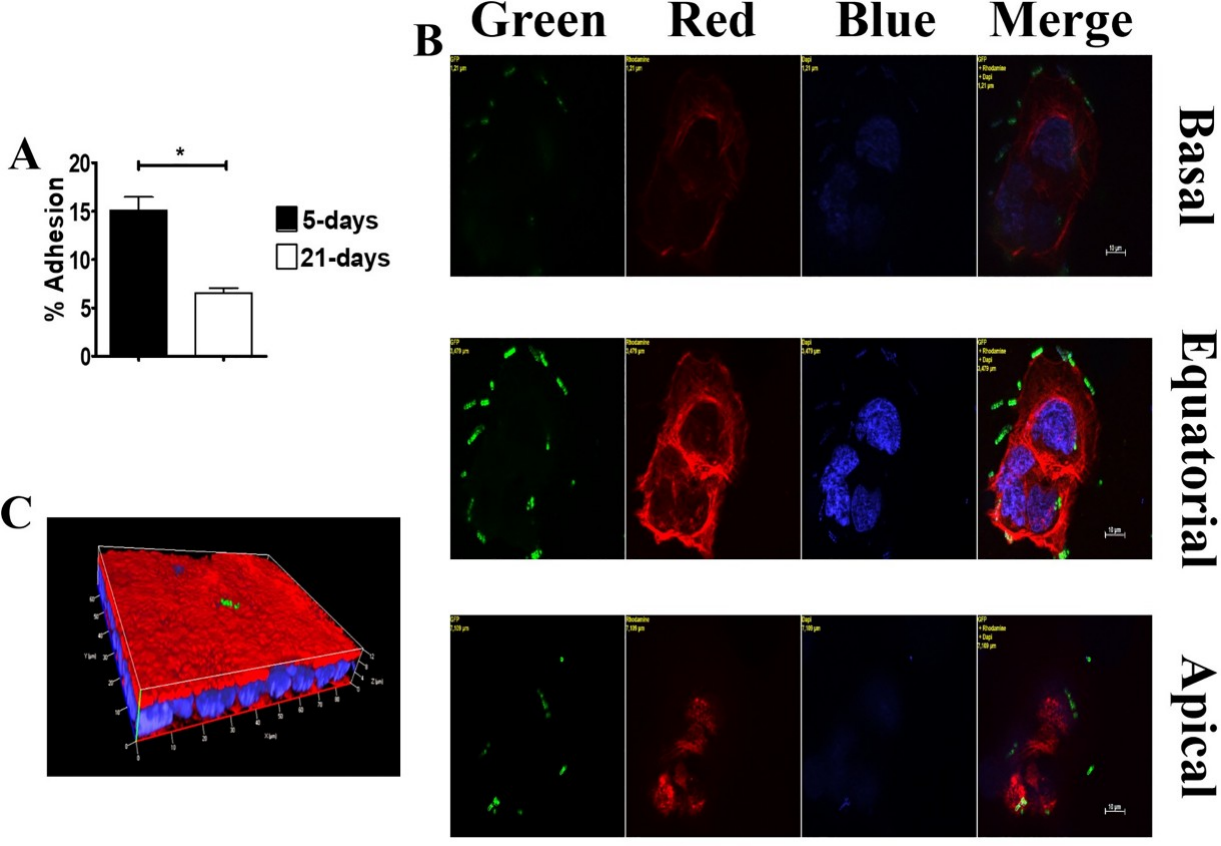
GBS adherence to Caco-2 cells. GBS adherence in infected islands and monolayers of Caco-2 cells. (**A)** GBS adherence as measured by CFU counting in 5-day-old islets and 21-day-old monolayers. Shown are means ± SD of three independent experiments conducted in triplicate. *, p<0.05 by Mann-Whitney test. (**B**) GBS adherence to the outer edges of 5-day-old small islets. Yellow labels indicate the filter(s) used and the distance, in μm, of the optical section from the slide plane. (**C**) GBS adherence to a small area of the brush border in 21-day-old monolayers (three-dimensional reconstruction). Nuclei and actin filaments were labeled with DAPI (Blue) and Phalloidin-iFluor 555 (Red), respectively. Bacteria (Green) were labeled using a rabbit anti-GBS serum followed by Alexa Fluor 488-conjugated anti-rabbit IgG. Scale bar = 10μm.

Growing Caco-2 cells are found in one of three aggregation types, depending on the age of cultures: 1) groups of few undifferentiated cells early after seeding; 2) islands of partially differentiated and polarized cells at approximately 5 days after the beginning of culture (5-day-old islets; **panel S1A, S1C and S1E Fig**); 3) confluent monolayers of fully differentiated and polarized cells showing the formation of “domes”, consisting of fluid accumulations beneath the monolayer surface (21-day-old monolayers; **panel S1B, S1D and S1E Fig)** [19, 20]. In initial experiments, we analyzed bacterial adherence using colony counts to determine the numbers of bacteria associated with 5- and 21-day-old cultures. Strikingly, the number of adhering bacteria was considerably higher in 5- than in 21-day-old cultures (**Fig 1A**). To study the mechanisms underlying these differences, bacterial adherence was analyzed by structured-illumination confocal immunofluorescent microscopy. In 5-day-old islets, a major portion of adherent bacteria was found along the outer edges of the islands, corresponding to the lateral surfaces of polarized cells (**Fig 1B**). GBS were also found in the intercellular spaces of large 5-day-old islands, particularly in optical sections corresponding to the parabasal levels of the vertical cell axis (**S2 Fig**). In contrast, in 21-day-old monolayers, only small numbers of bacteria were visible, and these colocalized to apical, but not subapical, sections (**Fig 1C**). These results indicate that hypervirulent GBS adheres preferentially to the lateral surfaces of Caco-2 cells at subapical and parabasal sites along the vertical cell axis. GBS adhered poorly to mature Caco-2 monolayers, in which subapical sites were not apparently exposed to the external environment. This adherence pattern is reminiscent of that of some enteroinvasive pathogens [11, 12, 21].

### GBS invasion occurs preferentially from the lateral surface of enterocytes

**Fig 2:**
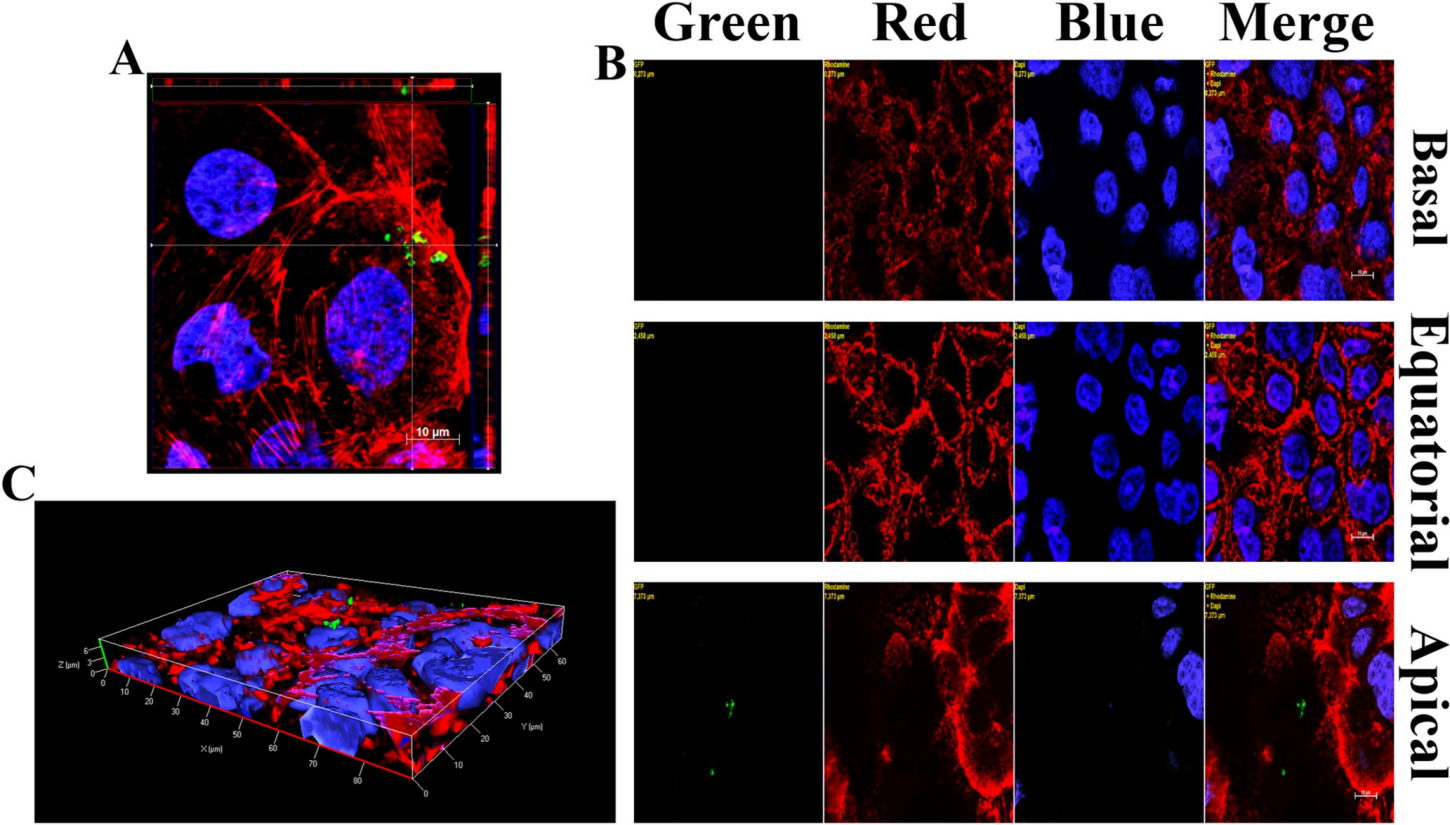
GBS invasion of 5- and 21-day-old Caco-2 cells. Fluorescence microscopy was performed on infected Caco-2 cells after killing extracellular bacteria with antibiotics. (**A**) Equatorial section in the z-stack of a 5-day-old islet with orthogonal views from x/z and y/z planes. (**B**) 21-day-old monolayer: few internalized GBS (Green) are visible in the apical section of the z-stack. Yellow labels indicate the filter(s) used and distance, in μm, of the optical section from the slide plane. (**C**) 3D reconstruction of a 21-day-old monolayer shown in panel B with few internalized GBS (green) under the apical surface. DAPI (Blue) and Phalloidin-iFluor 555 (Red) were used for labeling nuclei and actin filaments, respectively. GBS (Green) were visualized with a rabbit anti-GBS serum followed by Alexa Fluor 488-conjugated secondary antibody. Scale bar = 10μm.

**Fig 3:**
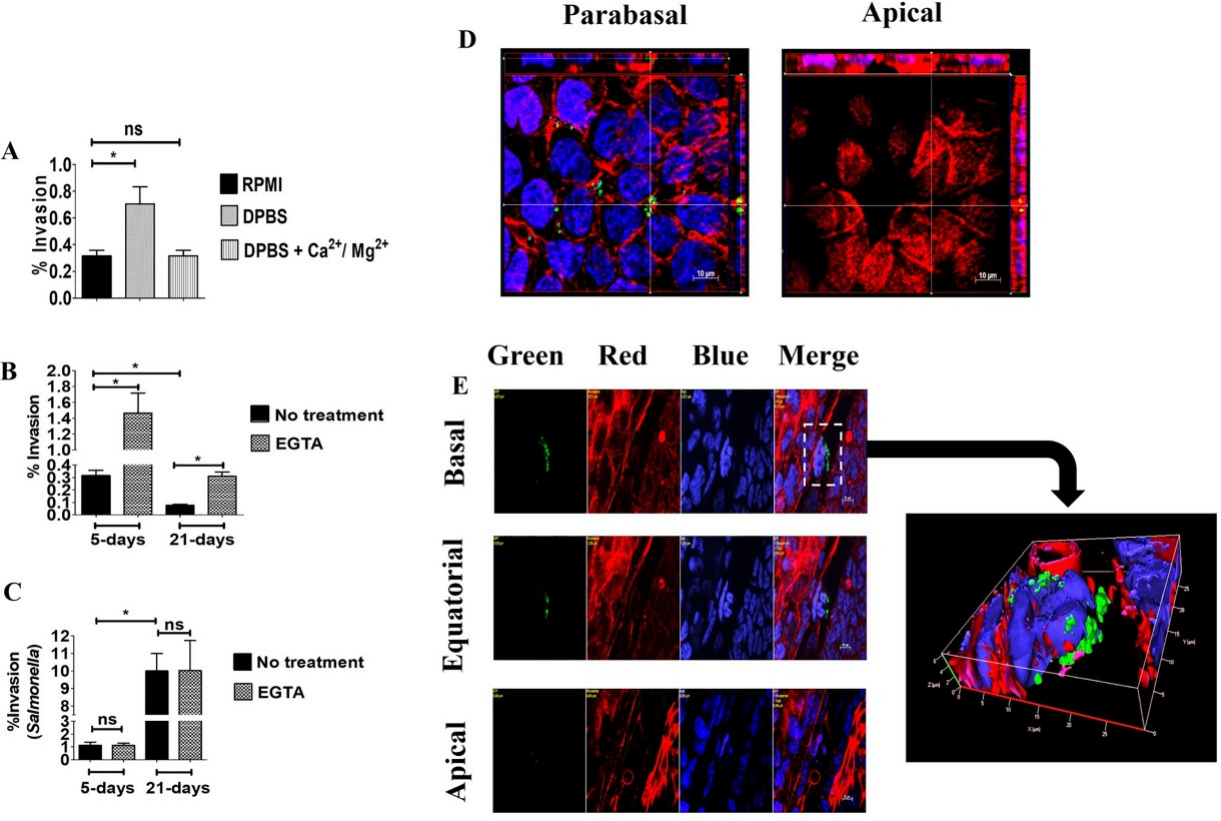
Enhanced GBS invasion of Caco-2 cells after disruption of intercellular junctions. Five-day-old islets and 21-day-old monolayers of Caco-2 cells were infected with GBS and extracellular bacteria were killed with antibiotics. Prior to infection, cells were treated as indicated to disrupt intercellular junctions. (**A**) Effects of Ca^2+^ and Mg^2+^ on GBS invasion of 5-day-old islets, as measured by CFU counts in cell lysates. DPBS, Dulbecco’s PBS without Ca^2+^ and Mg^2+^. (**B**) Effect of EGTA on GBS invasion, as measured by CFU counts in cell lysates. (**C**) Effect of EGTA treatment of Caco-2 cells on *S*. *enterica* var. Typhymurium (strain M20) invasion, as measured by CFU counts in cell lysates. Values shown in **A**, **B** and **C** are means ± SD of three independent experiments conducted in triplicate. *, p<0.05 by Mann-Whitney test. (**D**) Evidence of numerous invading streptococci (Green) in the parabasal region of a 5-day-old islet treated with EGTA. Orthogonal views from x/z and y/z planes are depicted laterally to show the tridimensional location of bacteria. (**E**) Bacteria interacting with the basal region of 21-day-old monolayers after EGTA treatment. Yellow labels indicate the filter(s) used and the distance, in μm, of the optical section from the slide plane. On the right is a three-dimensional reconstruction centered in the area indicated by the dashed rectangle. Bacteria (Green) were stained with an anti-GBS rabbit serum, followed by Alexa Fluor 488-conjugated anti-rabbit IgG. Actin (Red) and nuclei (Blue) were stained with Phalloidin-iFluor 555 and DAPI, respectively. Scale bar = 10μm.

The preferential adherence of bacteria to lateral surface of Caco-2 cells suggested that GBS might invade intestinal epithelial cells from this location. This seemed to be the case, since internalized GBS were found beneath the basolateral membranes in the cells of 5-day-old islands (**Fig 2A**). As expected, few internalized bacteria were detected in 21-day-old monolayers and these bacteria were localized under the apical cell surface (**Fig 2B and 2C**). Next, it was of interest to ascertain whether the presence of cell-to-cell junctions prevent GBS access to invasion-permissive sub-apical cell surfaces in monolayers. To this end, epithelial cell junctions were temporarily disrupted by treating cells with medium lacking Ca^2+^ [22, 23]. This treatment led to a marked increase in bacterial invasion compared to cells treated with a buffer supplemented with calcium and magnesium using both 5- and 21-day-old cultures (**Fig 3A and S3C Fig**). Another method to disrupt cell-to-cell junctions involves treatment with EGTA, a Ca^2+^- selective chelating agent. When cells were treated with EGTA, the ability of GBS to adhere to or invade 5- or 21-day-old Caco-2 cultures was considerably increased (**Fig 3B, S3A and S3B Fig**). To ensure that the ability of EGTA to increase GBS invasiveness was not related to non-specific effects, we used as control *Salmonella enterica* serovar Typhimurium strain M20, which is able to penetrate Caco-2 cells from the apical side, disrupting large areas of the brush border [24]. Unlike GBS, EGTA treatment did not induce any significant difference in *Salmonella enterica* serovar Typhimurium invasion of 5-day-old or 21-day-old Caco2 cells (**Fig 3C**). To ascertain whether GBS actually interact with basolateral cell surfaces after disruption of intracellular junctions, we analyzed EGTA-treated cultures by immunofluorescence microscopy. A considerable number of bacteria was visualized in the basolateral aspect of polarized cells in 5-day-old islands (**Fig 3D**) and 21-day-old monolayers (**Fig 3E**). Collectively, these data indicate that GBS can invade polarized Caco-2 cells with great efficiency through the lateral surfaces of the basal pole after disruption of intercellular junctions.

### GBS internalization involves caveolar lipid rafts

**Fig 4:**
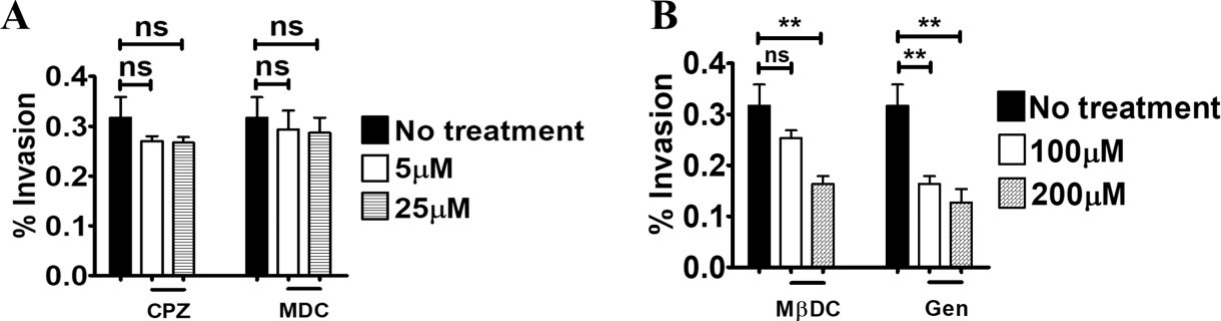
Lipid raft- and caveolin-dependent GBS entry into intestinal cells. Inhibitory effects of endocytosis-disrupting agents on GBS invasion of 5-day-old Caco-2 islets. Cells were infected with GBS in presence or absence of the inhibitors and intracellular bacteria were enumerated by CFU counts in cell lysates after killing extracellular bacteria with antibiotics. (**A**) Effect of chlorpromazine (CPZ) and monodansylcadaverine (MDC) at the non-cytotoxic concentrations of 5μM and 25μM, respectively. (**B**) Effect of inhibition of cholesterol-rich domains and tyrosine kinase on GBS internalization by methyl-betacyclodextrin (MβDC) or genistein (Gen) at the non-cytotoxic concentrations of 100μM and 200μM, respectively. Shown are means ± SD of three independent experiments conducted in triplicate. **, p<0.01 by one-way Anova and Bonferroni test.

A wide variety of pathogens invade epithelia *via* microrganism-directed endocytosis, which can be divided in different classes according to the molecular mechanisms involved [25–27]. We focused here on clathrin-, caveolae- and lipid raft-dependent endocytosis, since the majority of pathogenic bacteria use these internalization mechanisms [28–31]. Clathrin-mediated endocytosis can be inhibited by number of agents, including monodansylcadaverine (MDC) and chlorpromazine (CPZ), which reduce clathrin expression at the cell surface. Pretreatment with MDC or CPZ of 5- day-old Caco-2 cell cultures did not affect invasion at any of the tested doses, excluding a major role of chlatrin-mediated internalization in GBS invasion of Caco-2 cells (**Fig 4A**). Lipid rafts are cholesterol-rich membrane domains performing different functions, including signal transduction and endocytosis [31]. We assessed the role of lipid rafts in GBS internalization using the oligosaccharide methyl-β-cyclodextrin (MβCD), which decreases membrane cholesterol levels and blocks lipid raft functions. Pretreatment of 5- or 21-day-old Caco-2 cultures with MβDC significantly decreased GBS invasion, suggesting a role of lipid rafts in GBS internalization (**Fig 4B and S4 Fig**). Since lipid rafts can be divided into caveolae-containing and non-caveolar rafts, we examined the role of caveolin in GBS internalization. To this end, we pretreated Caco-2 cells with genistein, an inhibitor of tyrosine kinases involved in caveolae-dependent internalization. Genistein treatment significantly reduced GBS invasion in 5-day-old cultures, indicating that caveolin-dependent endocytosis is at least partially responsible for GBS internalization (**Fig 4B and S4 Fig**). Collectively, these data indicated that GBS invade cultured enterocyte-like cells by inducing caveolar lipid raft-dependent endocytosis.

**Fig 5:**
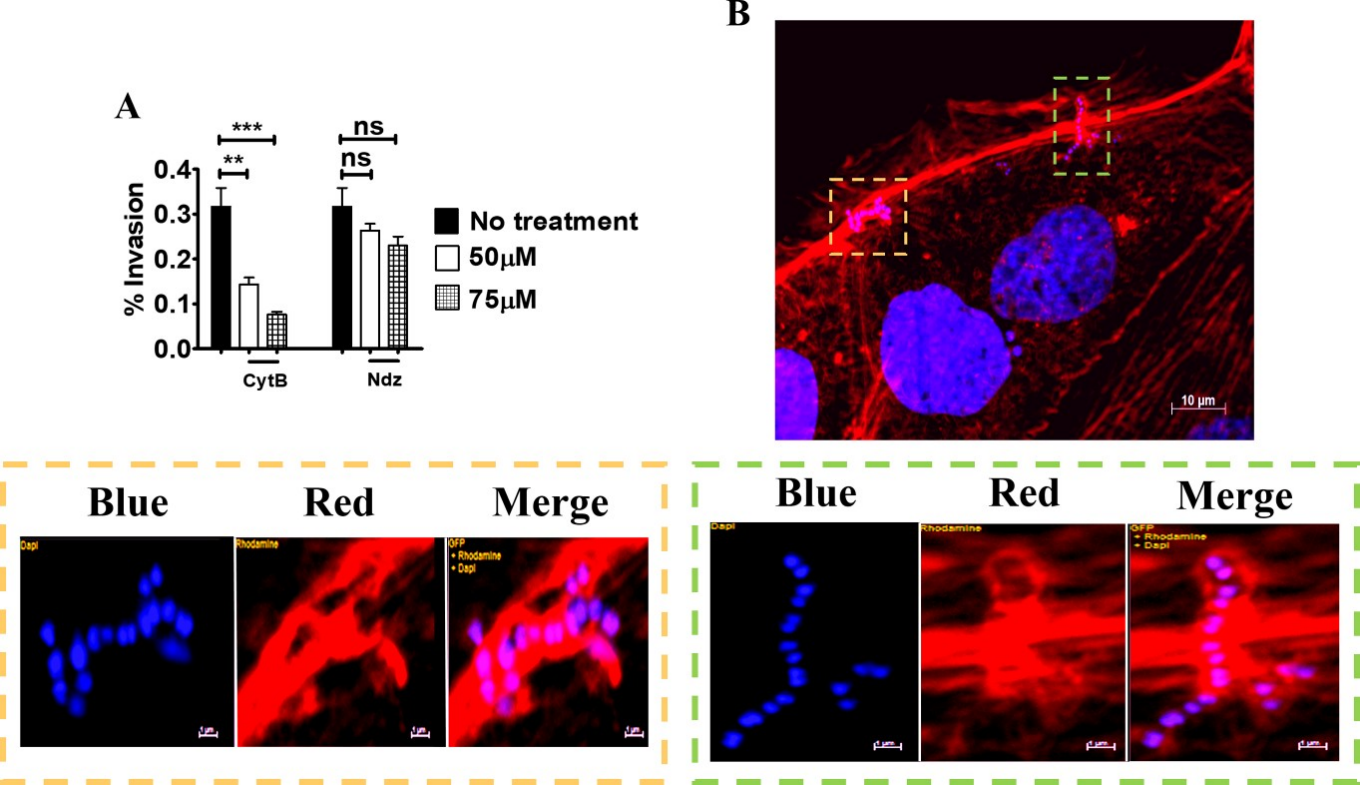
Actin-dependent invasion of Caco-2 cells by GBS. (**A**) Effect of cytochalasin B (CytB) and nocodazole (Ndz) on GBS invasion of 5-day-old Caco-2 islets at the non-cytotoxic concentrations of 50 and 75μM, respectively. Shown are means ± SD of three independent experiments conducted in triplicate **, p<0.01; ***, p<0,001 by one-way Anova and Bonferroni test. (**B**) Fluorescence microscopy analysis of 5-day-old islets infected with GBS. Scale bar = 10μm. The bottom panels are magnifications of the areas indicated by the two coloured dashed rectangles (orange and green). Cell-associated streptococci appear to be enveloped by actin filaments (red) below the outer edges of the cells. Scale bar = 1μm. Nuclei and nucleoids were stained with DAPI (Blue), actin with Phalloidin-iFluor 555 (Red).

Bacterial internalization normally requires host cytoskeleton rearrangements involving microfilaments, microtubules or both [32–34]. To examine whether the cytoskeleton is required for GBS invasion, Caco-2 cells were treated with cytochalasin B and nocodazole, which specifically inhibit actin filaments and microtubules, respectively. Treatment with cythocalasin B and, to a lesser extent, with nocodazole significantly decreased invasion of BM110 GBS, compared to control cells (**Fig 5A**). Moreover, immunofluorescence microscopy indicated that at 30 minutes post-infection GBS strongly interact with actin filaments, as indicated by colocalization between bacteria and phalloidin-stained actin at cell entry sites (**Fig 5B**).

### GBS trafficking and survival inside intestinal epithelial cells

**Fig 6:**
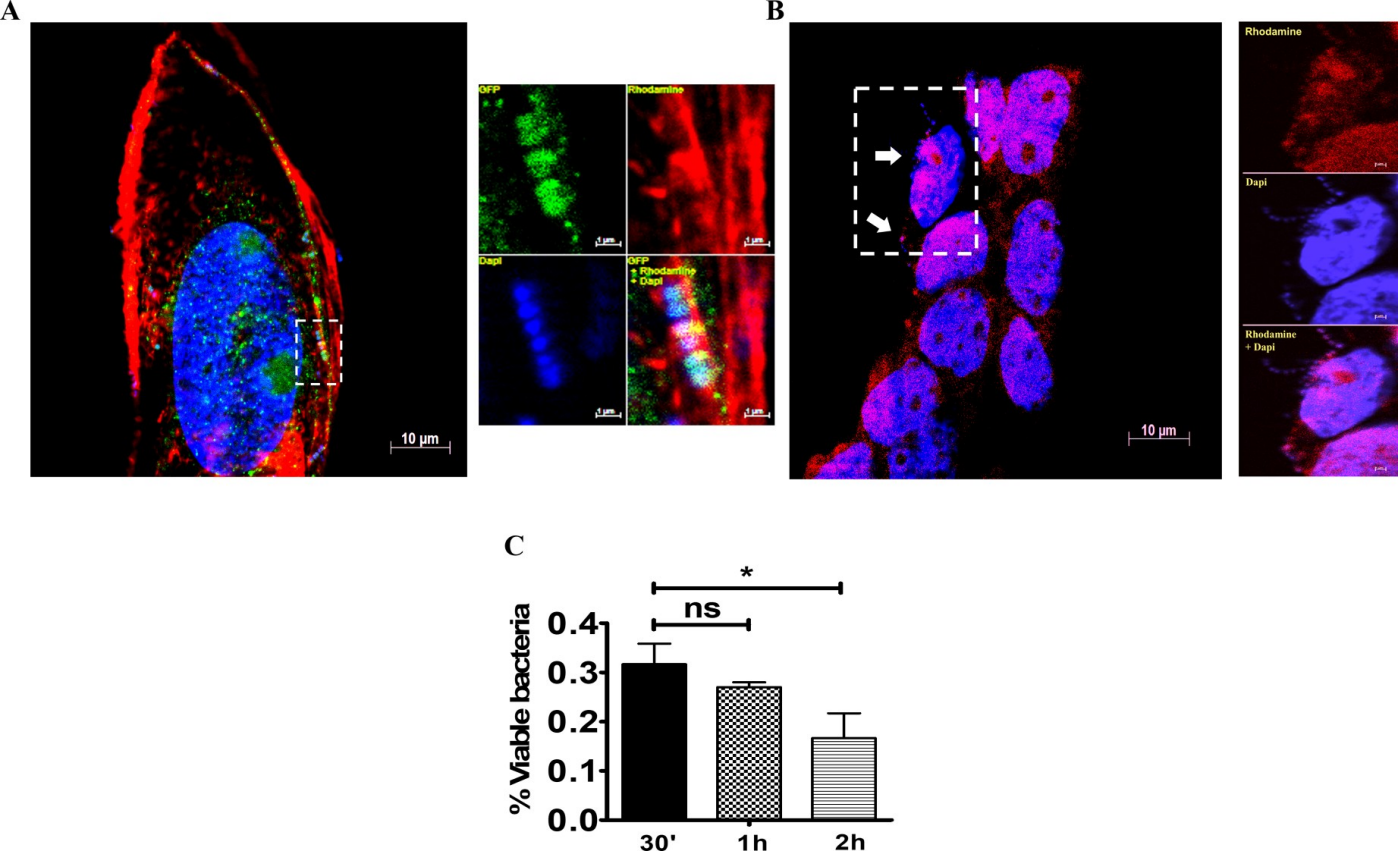
Colocalization of GBS with endosomal markers. Five-day-old islets were infected with GBS and stained for endosomal markers. **(A)** Co-localization of invading streptococci (Blue) with the early endosomal marker EEA-1 (Green) and actin filaments (Red) just below the cell membrane at 30 min after infection. Scale bar = 10μm. EEA-1 was stained using rabbit polyclonal antibodies followed by Alexa Fluor 488-conjugated anti-rabbit IgG. The right panels are magnifications of the area indicated by the dashed rectangle. Scale bar = 1μm. **(B)** Colocalization of GBS (Blue) with acidic endosomes (Red) at 2 h post-infection. White arrows indicate bacteria. The right panels are magnifications of the area indicated by the dashed rectangle. Scale bar = 1μm. **(C)** CFU counts in cell lysates at different times after treatment of 5-day-old Caco-2 islets with antibiotics to kill extracellular bacteria. Shown are means ± SD of three independent experiments conducted in triplicate. *, p<0.05; by one-way Anova and Bonferroni test.

We next investigated the intracellular fate of invading bacteria using immunofluorescence microscopy. At 30 min post-infection, GBS colocalized with EEA-1, a marker of early endosomes (**Fig 6A**). At later times (*i*.*e*. at 1 and 2 h post-infection) GBS predominantly resided in acidified vacuoles, as shown by co-localization with lysotracker, an acidotropic fluorescent die (**Fig 6B**). Bacterial viability slowly decreased after internalization, although the majority of bacteria were still viable at two hours post-infection (**Fig 6C**).

## Discussion

GBS CC-17, which is strongly associated with LOD, has increased capacities for late colonization of the infant gut, raising the possibility that the intestinal tract is a port of bacterial entry in the pathogenesis of this disease [9, 10]. In humans, the intestinal epithelium performs not only digestive and absorptive functions, but also acts as an extremely efficient physical barrier against pathogens [35, 36]. This barrier function rests, to a great extent, on the integrity of apical-basal polarity and junctional complexes, including tight junctions, adherence junctions, focal adhesions and hemidesmosomes [36–38]. For these reasons, we chose to use, as a model system for the intestinal epithelium, Caco-2 cells, which undergo over time differentiation changes leading to the formation of cell-to-cell junctions, apical brush borders and enterocyte-like morphology [14, 39]. Moreover, to study GBS invasion, we used the prototypical BM110 strain, which is representative of the CC17 clonal complex and displays a unique set of adhesins and virulence factors, comprising both CC17-specific cell wall proteins and adhesins expressed by multiple clonal complexes [9, 18, 40, 41]. The major finding of the present study is that GBS can adhere to and invade enterocytes from their basolateral surfaces in polarized cells. In contrast, invasion from the apical surface was a rare event. *S*. Typhimurium displayed an opposite behaviour and invaded Caco-2 cells from their apical but not lateral surfaces, confirming the specificity of our findings. The ability of GBS to preferentially invade the lateral surface of enterocytes is shared by *Listeria monocytogenes* [11], *Shigella flexneri* [12] and *Campylobacter jejuni* [13], but not by other enteropathogens, such as *Salmonella* spp. or *E*.*coli* [24, 42].

In the present study, GBS invaded Caco-2 cells with great efficiency when cell-to-cell junctions were disrupted by Ca^2+^ depletion, but not using intact monolayers. Our data suggest that the GBS receptors on intestinal cells are selectively distributed in the lateral surface of the basal cell pole and are at least partially hidden in intact monolayers. This raises the question as to how GBS can gain access to their receptors when cell-to-cell junctions are intact in the normal mucosa. In this respect, at least two possibilities can be envisioned. It is likely, in the first place, that GBS temporally affect the integrity of cell-to cell junctions. This possibility is supported by observations that GBS intimately associate with and transiently open tight junctions in monolayers of cervical and Caco-2 cells, as shown by transmission electron microscopy [43]. Accordingly, in the present study, GBS were often found along the lateral surface of partially differentiated cells. In the second place, cell-to-cell contacts loosen up to allow passage of cells and fluids in the intestinal lumen under inflammatory conditions. Although GBS carriage is not commonly regarded as being accompained with innate immune responses, recent data point to the occurrence of inflammatory phenomena during colonization, at least in the vaginal habitat [44–46]. Elevated levels of IL-1α were detected in GBS-positive non-pregnant women [44]. Moreover, increased production of other pro-inflammatory cytokines and neutrophil accumulation were documented in mouse models of vaginal colonization [46]. Future studies should assess whether GBS colonization of the gut is accompanied with an innate immune responses and should establish the impact, if any, of such responses on the barrier function of the intestinal epithelium.

Of course, our data showing invasion from the lateral surfaces of enterocytes do not exclude the existence of other mechanisms for invasion of the intestinal barrier by GBS. It has been shown, for example, that this pathogen can use a paracellular route to transverse Caco-2 monolayers growing on filters [43]. Moreover, CC17 GBS can apparently cross the mouse intestinal barrier using M cells, which are specialized intestinal epithelial cells with a marked ability to sample luminal contents and deliver it to the underlying mucosal tissues [47]. Indeed, transcytosis through M cells has been shown to occur *in vivo* for *Shigella* and other enteropathogens [48]. Therefore, it appears that GBS might invade the intestinal barrier through multiple pathways. An important point to be addressed in future studies will concern the nature of the GBS receptor that mediates preferential invasion through the lateral surface of enterocytes. A number of potential bacterial receptors are known to be expressed at specific locations on the cell membrane of these cells. E-cadherin is restricted to the cell-cell border of intestinal epithelial cells [49] and acts as a ligand for listerial internalin A, which can explain the preferential internalization of *L*. *monocytogenes* at the lateral enterocyte surface [50]. The α_5_β_1_ integrin, which can act as a receptor for CC17 GBS in brain endothelial cells [51], is also found on the lateral surfaces of enterocytes and on the apical surfaces of M cells [52]. Studies are underway to ascertain if CC17 uses the α_5_β_1_ integrin and other integrins to invade the lateral surface of enterocytes and cross the intestinal barrier.

In the present study, adherence of GBS to the lateral surfaces of Caco-2 cells was followed by caveolin- and lipid raft-dependent endocytosis, while clathrin-mediated internalization was not apparently involved. Similar to GBS, a number of other pathogens, such as *Streptococcus uberis*, *Chlamydia trachomatis* and *Pseudomonas aeruginosa*, were reported to invade epithelial through caveolae-dependent mechanisms [53–56]. We show here that caveolar lipid raft-dependent endocytosis results in close association of GBS with actin filaments and internalization in EEA1-positive early endosomes, followed by their rapid passage into lysotracker-positive, acidified compartments. This localization resembles the classical endosomal pathway used by many pathogens to reside more or less temporarily in intestinal cells [56, 57]. In conclusion, we demonstrate that GBS can invade polarized enterocytes through an actin- and caveolar lipid raft-dependent mechanism after adhering to the basolateral portion of the cell surface. Future studies should assess the relative importance of this invasion pathway *in vivo*. These data may be useful to develop alternative strategies to prevent LOD by blocking GBS invasion of the intestinal barrier.

## Supporting information

S1 Fig Morphological features of 5-day- and 21-day-old Caco-2 cells. **(A)** Cellular islands of different dimensions at 5 days post-seeding (100x, optical microscopy). **(B)** “Dome” structures in completely differentiated monolayers at 21 days post-seeding (200x, optical microscopy). **(C)** Fluorescence microscopy analysis of 5-day-old islands. Left panels show an islet consisting of few cells, while right panels show a larger island. Microvilli are evident in the right lower panel. **(D)** Fluorescence microscopy analysis of 21-day-old monolayers with a well established dotted brush border. Nuclei and actin filaments were labeled with DAPI and Phalloidin-iFluor 555, respectively. Fluorescence microscopy was performed using a Zeiss Observer.Z1 microscope with an Apotome apparatus and were acquired at the z-stack positions shown in **(E)**. Scale bar = 10μm.
(TIF)

S2 Fig GBS cell adherence in a large 5-day-old island. Fluorescence microscopy analysis of an infected 5-day-old island. The top left panel was obtained by integrating 18 optical sections taken at various distances along the z axis using the Extended Focus (EF) module of the AxioVision software. The other panels represent single optical sections taken at the indicated levels along the z axis. Orthogonal views from x/z and x/y planes are also shown. Yellow labels indicate the distance of the optical section from the slide plane. Scale bar = 10μm. Nuclei and nucleoids were stained with DAPI (Blue), actin with Phalloidin-iFluor 555 (Red) and bacteria with rabbit anti-GBS serum followed by an Alexa Fluor 488-conjugated anti-rabbit IgG (Green).
(TIF)

S3 Fig Weakening of intercellular junctions enhances GBS interactions with Caco-2 cells. Effect of EGTA treatment of 5- (**A**) or 21-day-old (**B**) Caco-2 cell cultures on GBS adherence as measured by CFU counts. **C**) Effect of cell treatment with media with or without Ca^2+^ and Mg^2+^ on GBS invasion using 21-day-old monolayers, as measured by CFU counts in cell lysates. DPBS, Dulbecco’s PBS without Ca^2+^ and Mg^2+^. Shown are means + SD of three independent experiments conducted in triplicate. *, p<0.05 by Mann-Whitney test.
(TIF)

S4 Fig GBS internalization in EGTA-treated cells from 21-day old monolayers involves lipid raft- and caveolin-dependent mechanisms. Inhibitory effects of endocytosis-disrupting agents on GBS cell invasion in 21-day-old monolayers. Prior to infection, cells were treated with EGTA to disrupt intercellular junctions. Infection was performed in presence or absence of the inhibitors and intracellular bacteria were enumerated by CFU counts in cell lysates after killing extracellular bacteria with antibiotics. Shown are the effects on GBS internalization of inhibition of cholesterol-rich domains and tyrosine kinase by, respectively, methyl-betacyclodextrin (MβDC) or genistein (Gen) at the non-cytotoxic concentrations of 100 μM and 200 μM, respectively. Shown are means ± SD of three independent experiments conducted in triplicate. *, p<0.05; **, p<0.01 by one-way ANOVA and Bonferroni test.
(TIF)
