# Supplementary figures and images for: Invasion and trafficking of hypervirulent group B streptococci in polarized enterocytes
Source: PLoS One. 2021 Jun 15;16(6):e0253242. doi: 10.1371/journal.pone.0253242 (PMC8205152; doi:10.1371/journal.pone.0253242)

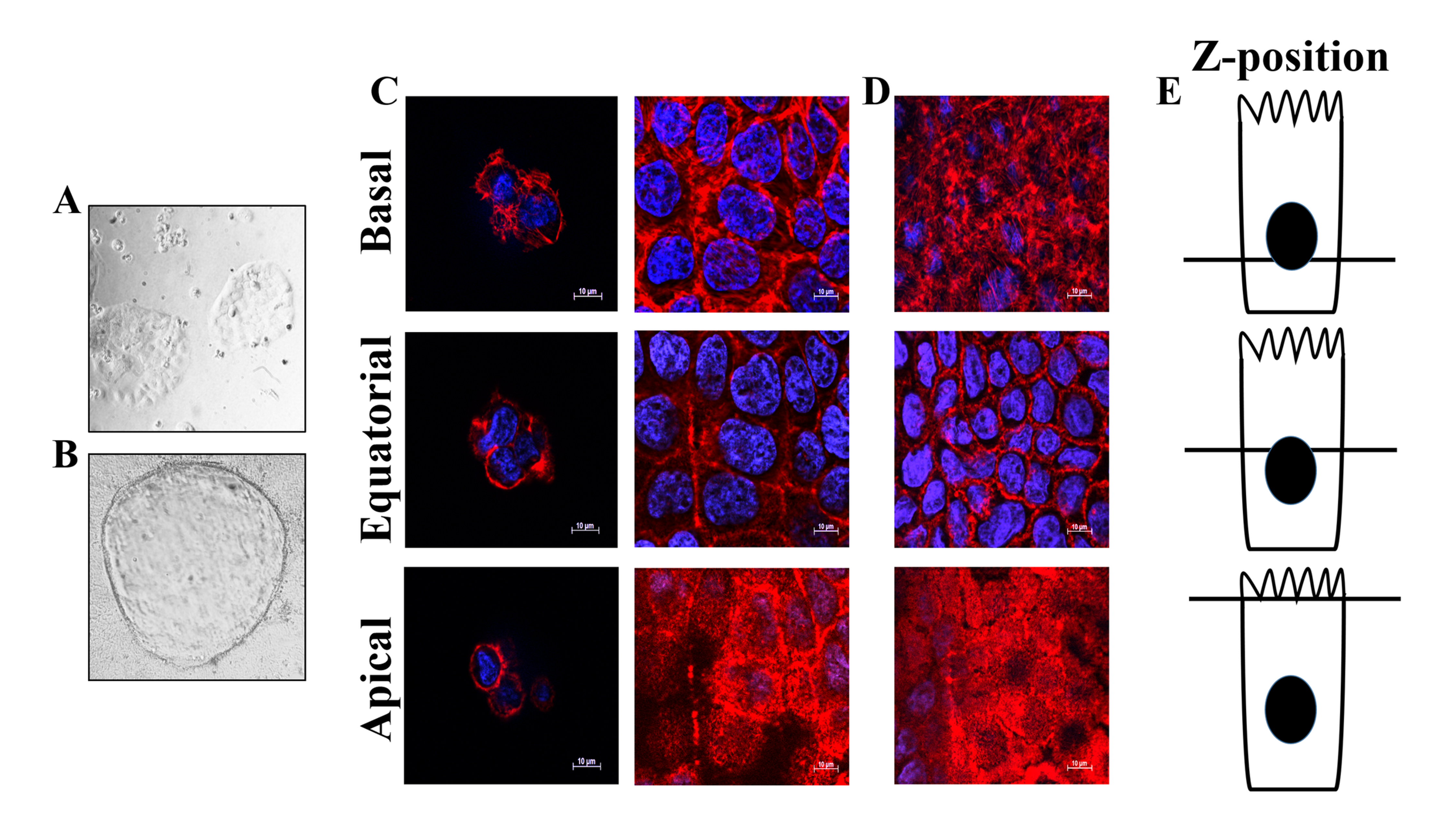

Supplement: S1 Fig — (A) Cellular islands of different dimensions at 5 days post-seeding (100x, optical microscopy). (B) “Dome” structures in completely differentiated monolayers at 21 days post-seeding (200x, optical microscopy). (C) Fluorescence microscopy analysis of 5-day-old islands. Left panels show an islet consisting of few cells, while right panels show a larger island. Microvilli are evident in the right lower panel. (D) Fluorescence microscopy analysis of 21-day-old monolayers with a well established dotted brush border. Nuclei and actin filaments were labeled with DAPI and Phalloidin-iFluor 555, respectively. Fluorescence microscopy was performed using a Zeiss Observer.Z1 microscope with an Apotome apparatus and were acquired at the z-stack positions shown in (E). Scale bar = 10μm. (TIF) [file pone.0253242.s001.tif]

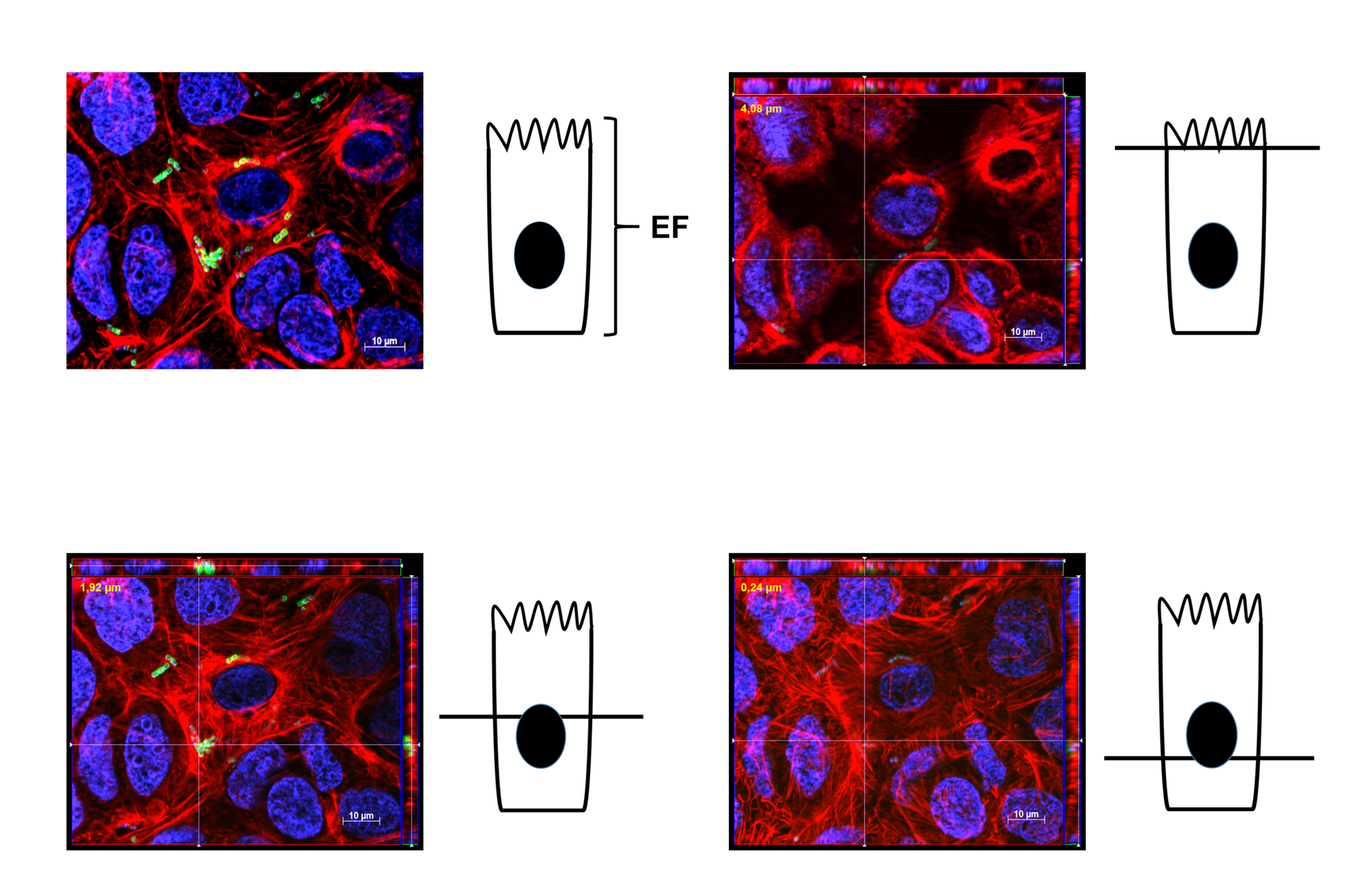

Supplement: S2 Fig — Fluorescence microscopy analysis of an infected 5-day-old island. The top left panel was obtained by integrating 18 optical sections taken at various distances along the z axis using the Extended Focus (EF) module of the AxioVision software. The other panels represent single optical sections taken at the indicated levels along the z axis. Orthogonal views from x/z and x/y planes are also shown. Yellow labels indicate the distance of the optical section from the slide plane. Scale bar = 10μm. Nuclei and nucleoids were stained with DAPI (Blue), actin with Phalloidin-iFluor 555 (Red) and bacteria with rabbit anti-GBS serum followed by an Alexa Fluor 488-conjugated anti-rabbit IgG (Green). (TIF) [file pone.0253242.s002.tif]

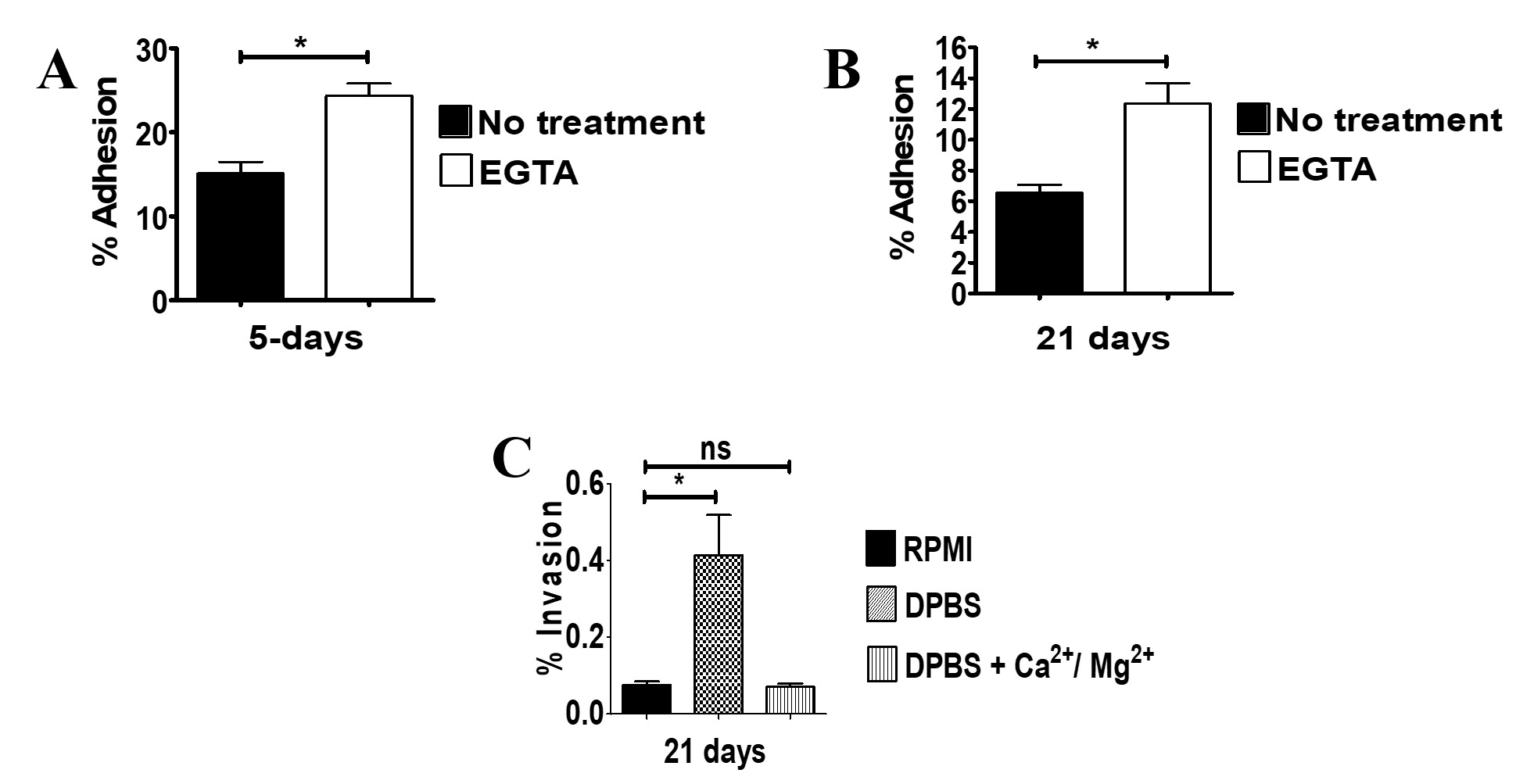

Supplement: S3 Fig — Effect of EGTA treatment of 5- (A) or 21-day-old (B) Caco-2 cell cultures on GBS adherence as measured by CFU counts. C) Effect of cell treatment with media with or without Ca2+ and Mg2+ on GBS invasion using 21-day-old monolayers, as measured by CFU counts in cell lysates. DPBS, Dulbecco’s PBS without Ca2+ and Mg2+. Shown are means + SD of three independent experiments conducted in triplicate. *, p<0.05 by Mann-Whitney test. (TIF) [file pone.0253242.s003.tif]

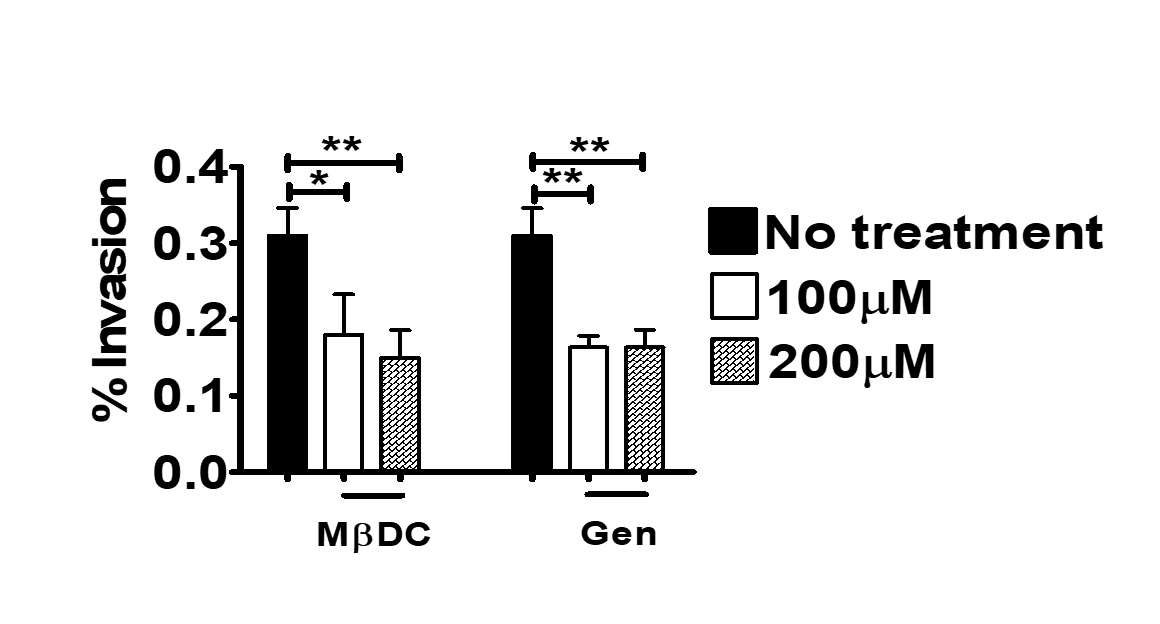

Supplement: S4 Fig — Inhibitory effects of endocytosis-disrupting agents on GBS cell invasion in 21-day-old monolayers. Prior to infection, cells were treated with EGTA to disrupt intercellular junctions. Infection was performed in presence or absence of the inhibitors and intracellular bacteria were enumerated by CFU counts in cell lysates after killing extracellular bacteria with antibiotics. Shown are the effects on GBS internalization of inhibition of cholesterol-rich domains and tyrosine kinase by, respectively, methyl-betacyclodextrin (MβDC) or genistein (Gen) at the non-cytotoxic concentrations of 100 μM and 200 μM, respectively. Shown are means ± SD of three independent experiments conducted in triplicate. *, p<0.05; **, p<0.01 by one-way ANOVA and Bonferroni test. (TIF) [file pone.0253242.s004.tif]
